# Ewes Direct Most Maternal Attention towards Lambs that Show the Greatest Pain-Related Behavioural Responses

**DOI:** 10.1371/journal.pone.0134024

**Published:** 2015-07-28

**Authors:** Agnieszka Futro, Katarzyna Masłowska, Cathy M. Dwyer

**Affiliations:** 1 Royal (Dick) School of Veterinary Medicine, University of Edinburgh, Edinburgh, United Kingdom; 2 SRUC (Scotland’s Rural College), Edinburgh, United Kingdom; ETH Zurich, SWITZERLAND

## Abstract

Although neonatal farm animals are frequently subjected to painful management procedures, the role of maternal behaviour in pain coping, has not been much studied. We investigated whether ewes were able to distinguish between lambs in pain and those that were not, and whether their behaviour altered depending on the severity of lamb pain. Eighty male lambs were allocated to one of 4 pain treatments within 24 hours of birth. Lambs were either handled only (C), bilaterally castrated with tight rubber rings (RR), as for RR but with the application of a Burdizzo clamp immediately proximal to the ring (Combined) or subjected to short scrotum castration (SSC) where the testicles were retained within the abdomen and only the scrotum removed. The behaviour of the ewe, treated lamb and untreated sibling where present (n = 54) were recorded for 30 minutes after treatment. Castration treatment increased the expression of abnormal standing and lying postures, specific pain-related behaviours (head-turning, stamping/kicking, easing quarters, tail wagging) and composite pain scores (P<0.001 for all). The greatest expression of pain-related behaviours was shown by lambs in the RR group, which were the only group to show rolling responses indicative of severe pain, followed by the SSC group. Ewes expressed more licking/sniffing responses to the RR and SSC lambs than towards the Combined and C lambs (P<0.05), and oriented most to RR lambs and least to C lambs (P<0.001). Ewes with two lambs also directed more attention towards the treated than the untreated lamb (P<0.001). The quantity of maternal care directed towards the lamb was positively correlated with the expression of active pain behaviours. The data demonstrate that ewes are able to discriminate between lambs in pain and those that are not, and that their response is increased with a greater severity of pain.

## Introduction

Mammalian mothers play a fundamental role in the lives and survival of their young offspring, providing nutrition, thermal and physical protection and opportunities for social learning (e.g. as reviewed by [1]). In addition, mothers are a source of comfort and psychological protection, with evidence for attenuated stress and pain responsiveness in offspring receiving tactile stimulation or nursing contact from their mother (rats [2], humans [3]). Female rats express more licking and grooming towards their neonatal pups that were subjected to painful stimuli than those that were not [4,5]. These changes in maternal care were associated with altered pain sensitivity in the offspring, suggesting that mothers are able to buffer the pain experience of their offspring through increased maternal behaviour. Further, maternal separation or artificial rearing in rats is associated with increases in pain sensitivity in later life [6–8]. These data suggest that another function of maternal behaviour is to support offspring during adversity and prevent sensitisation of pain responses in adult life. However, the impact of maternal care on the pain responses of their young has received less attention in farm animals, although there is evidence that close maternal contact between ewe and lamb is correlated with increased nociceptive thresholds in the lamb [9]. Recent evidence also suggests that farm animals may also be affected by the sensitising effects of early pain exposure (e.g. tail-docking in sheep: [10]).

Young farm animals are frequently subjected to painful husbandry procedures. Tail docking in pigs and sheep, and castration in pigs, sheep and cattle are common on farm practices, often carried out without the use of anaesthesia or analgesia. In many countries castration of lambs is practised to prevent unwanted mating, reduce aggression and fighting between rams [11] and undesirable meat characteristics [12]. The most common method (e.g. used by more than 95% of UK sheep farmers who castrate lambs [13]) is the application of tight rubber bands to the neck of the scrotum causing ischaemia and tissue necrosis distal to the ring. Although cheap and effective, this method is associated with acute pain responses in the lamb for more than 2 hours after application [14,15], abnormal postures for up to 24 hours, increased activity for up to 6 days and responses to palpation for more than 9 days after application [16]. Other methods of castration include short scrotum castration, where the testicles are returned to the abdominal cavity and only the scrotum is removed, and use of a device or clamp (e.g. Burdizzo) to crush the nerves in the neck of the scrotum and the spermatic cords (sometimes combined with a rubber ring where the clamp is placed proximal to the ring), which result in less intense acute pain responses from the lamb [14].

As neonatal farm animals are frequently exposed to pain in early life, which may have longer term consequences for their responding to pain in adult life, the expression of maternal care may be affected by the early pain experience of her offspring. There is evidence to suggest that ewes direct more maternal care, in the form of sniffing, licking and glancing, towards their offspring subjected to painful procedures and expressing pain behaviours, compared to their untreated twins [17]. In this study we extend these observations by asking whether the maternal care expressed by the ewe is related to the pain experience of the lamb by comparing the maternal behaviour expressed by ewes when their lambs are subjected to different castration treatments, or handled only. We hypothesised that ewes would direct more maternal care towards lambs experiencing the castration treatment known to cause the greatest acute pain responses, i.e. rubber rings [14], in comparison to other treatments (short scrotum castration, Burdizzo), where lower pain response are seen and to a non-pain treatment (handling).

## Materials and Methods

### Animals and housing

This study involved 65 Mule (Scottish Blackface x Border Leicester) ewes mated to either Suffolk or Texel rams and their 80 male lambs, which were drawn from a larger flock of approximately 500 ewes. After mating ewes were scanned with ultrasound at approximately 70 days gestation to determine litter size and managed in groups of the same litter size thereafter. Ewes were brought indoors to large, straw-bedded pens (approximately 3 x 20 m, 30 ewes per pen) approximately 8 weeks before lambing and fed concentrate (locally milled ewe nuts, 218g crude protein per kg dry mass) to meet requirements for maintenance and foetal growth, with ad libitum access to hay and water. Ewes gave birth within the group pens but were moved to individual pens (approximately 1.5 x 1.5 m) shortly after the birth of all lambs (generally within 1 hour of lambing). Ewes and lambs were allocated to a treatment; balancing as described below, at approximately 12 hours after birth, after assessing that ewes were maternal towards their lambs and that lambs were sucking from their mothers unaided. Following allocation to trial, ewes and lambs were transported together to a separate shed (30 m x 20 m), where each ewe and her litter was placed into an individual pen (approximately 2 m x 2 m) where castrations and behavioural observations took place. Individual ewes were separated by open barred gates allowing visual, auditory and tactile contact between ewes. The pens were straw-bedded, and the shed had natural ventilation (slatted sides) and was maintained at ambient temperature (approximately 5–15°C). Once behavioural observations were complete ewes and lambs were moved to large group pens (approximately 10 ewes and associated lambs per pen, each pen 10 m x 10 m) for up to 24 hours and then moved outside to a paddock.

### Procedures

The 80 lambs were allocated to 1 of 4 treatment groups (n = 20 per group) as follows, with treatments applied between 24–48 hours old:

Control (C): Lambs were physically handled for approximately 1 minute in a similar manner to castrated lambs but a rubber ring was not placed around the scrotum.

Rubber ring castration (RR): Full bilateral castration with a conventional rubber ring. A rubber ring was placed around the neck of the scrotum using an elastrator to prevent blood flow to the scrotum and testes distal to the ring.

Short Scrotum Castration (SSC): The procedure was as described for RR except the testes were pushed back into the abdominal cavity and rubber rings were applied around the neck of the scrotum below the testes to prevent their descent (crytorchid). Strictly lambs were not castrated but made infertile by the treatment.

Rubber Ring Castration combined with a Burdizzo treatment (COMBINED): Lambs were castrated by application of novel tight rubber rings (developed as previously described,: although these rings have a smaller internal diameter than conventional rings the acute pain responses of lambs castrated with these rings did not differ from conventional, Molony et al., 2012) to the neck of the scrotum (as described for RR) followed immediately by crushing the nerves and spermatic cords with a Burdizzo (Big Nipper; Ritchey Ltd, Ripon, UK) proximal to the ring as previously described (Molony et al., 1993).

Each treatment group was balanced for litter size as single, twin and triplet born lambs were used, maternal parity (first or subsequent pregnancy), and sire breed as shown in Table 1. Although 4 lambs per treatment group were born as part of a triplet litter each ewe only raised a maximum of two lambs. Therefore, triplet litters were reduced to two lambs prior to castration treatments being applied, and the remaining lamb was either fostered or artificially reared separate to this study (following the standard husbandry procedures of the farm on which the study was conducted). One control lamb was born as part of a twin litter but his (female) twin died prior to allocation therefore this lamb was raised as a single. Within twin and triplet litters, if both lambs were male, lambs were allocated to the same treatment group (n = 15 litters, 30 lambs: 4 litters each to CRR, RRNC, SSC and 3 litters to C treatment) but castration treatments occurred separated by at least 2 hours when active pain behaviours are considered to have decreased to uncastrated baselines [14], although other abnormal responses may still be apparent [16]. All manipulations took place in the home pen and took between 1 and 3 minutes to perform. All lambs, whether castrated or untreated littermates, remained with their mothers in the same pen throughout.

**Table 1:** Distribution of lambs by birth and rearing litter size, dam parity and sire breed between treatment groups.

	C	RR	SSC	Combined	Total
**Birth litter** size					
1	3	3	3	3	12
2	13	13	13	13	52
3	4	4	4	4	16
**Rearing litter size**					
1	4	3	3	3	13
2	16	17	17	17	67
**Dam parity**					
1	7	6	8	7	28
2	3	5	1	3	12
3	4	5	8	5	22
4+	6	4	3	5	18
**Sire Breed**					
Texel	12	13	11	11	47
Suffolk	8	7	9	8*	32

*1 lamb not recorded for sire breed

At least 2 hours after castration of all lambs in a litter, and after behaviour recording had finished, lambs were tail-docked with rubber rings (to meet the requirements of the farm policy to prevent cutaneous myiasis) following injection of 0.3 mL 5% procaine with 0.002% adrenalin (Willcain, Dechra Veterinary Products Limited, Shropshire, UK) to provide local anaesthesia and ear tagged with electronic identification tags (Shearwell Data Ltd, Somerset, UK).

### Behavioural data collection

Prior to castration twin litters were identified by marking one lamb with a paint stripe (Ritchey Sprayline Stock marker, Ritchey Ltd, Ripon, UK) to facilitate video recognition. Continuous video records were made using a Canon XM2 3CCD Digital Video Camcorder (Canon Inc, Japan) mounted on a tripod above each pen positioned so that the whole pen was visible. Video records began immediately before lambs were castrated/sham castrated and continued for 30 minutes after the experimenter had left the pen. A previous study has shown that active pain behaviours following castration increase in frequency to reach a peak at 15 minutes following application of the ring before declining [14]. The timing of behavioural observation was designed to capture the period when lambs expressed the greatest amount of pain behaviour, rather than to include all expression of pain behaviours following castration.

All behavioural data were recorded by a single observer, using the Observer XT 9.0 (Noldus Information Technology, Wageningen, The Netherlands). An ethogram of lamb behaviours was developed based on the previous studies of pain in lambs following castration and tail docking [14,16,18] and is shown in Table 2. Lamb foot stamping, kicking and easing quarters were recorded together as it was difficult to distinguish between these activities on the videotape. The video record did not allow us to distinguish clearly between the lamb drinking milk or only nosing the udder region, therefore these behaviours were recorded as a single category of ‘teat seeking’. Tail wagging that was clearly associated with teat seeking was not recorded separately as tail wagging, which was restricted to wagging occurring away from the udder. Rolling was recorded as an indicator of very strong pain reactions e.g. [19, 20]. Post-hoc analysis of restlessness was performed by defining a number of times the lamb made postural transitions between lying and standing. The ethogram for ewe behaviours was based on previous studies [17,21] and shown in Table 2. The ewe behaviours of ‘head up’ (vigilance posture) and ‘agitation’ were analysed together as an ‘alert’ response. For ewes with two lambs, where only 1 lamb was considered to be in pain at each observation period, the teat seeking behaviours of both lambs, and the attention of the ewes (sniffing/licking and orientation) to both lambs were recorded.

**Table 2:** Definitions of lamb and ewe behaviours recorded in the first 30 min following different castration treatments or sham castration. Lamb behaviours, except postures and teat seeking, were recorded as events; ewe behaviours, lamb postures and teat seeking were recorded as states.

Behaviour	Definition
**Lamb Behaviour**	
Foot stamping/kicking/ease quarters	Lamb raises and moves hindlimb backwards or forwards without moving other limbs
Head turning	Head is moved beyond the shoulder along the flanks or to the scrotum
Tail wagging	Rapid side-to-side tail movements made when the lamb is not teat seeking; tail wagging bouts were recorded as events, a bout was considered to have ended when the tail had been stationary for 2 secs.
Teat seeking	Lamb has nose within 5 cm of the udder, nudging udder or with teat held in the mouth, tail may be wagging.
Abnormal ventral lying	Lamb lies on the sternum with hindlegs extended whilst keeping the scrotum lifted above the ground
Abnormal lateral lying	Lamb lies on the flank with the hind legs extended
Normal lying	Ventral recumbency, the lamb lying on its sternum and abdomen with legs tucked in or partly on its side with legs relaxed
Normal standing/walking	Lamb stands or walks without unsteadiness, hindlimbs parallel to forelimbs
Abnormal standing/walking	Lamb stands or walks unsteadily, with swaying or an arched back, hind limbs may be apart and positioned further back than normal
Rolling	Lamb rolls and kicks from side to side whilst lying laterally, may maintain position on the back with hindlegs extended for a period
Restlessness	Number of postural transitions between standing and lying
Active Pain Behaviour	Summation of foot-stamp/kick/ease quarters, head turning and rolling
Active Pain Behaviour + tail wag	As above but including tail wagging
REQ	Active Pain Behaviour +Restlessness
REW	REQ + tail wagging
**Ewe Behaviour**	
Sniffing/licking lambs (treated lamb (L1) and untreated sibling (L2) were distinguished)	Ewe moves her muzzle to within 5 cm of the lamb, accompanied by sniffing, nibbling or licking movements
Orientation (L1 or L2 distinguished)	Ewe directs head towards the lamb with ears forward and pointing towards lamb
Head up (vigilance)	Ewe stands immobile with head above the level of the shoulders, ears pointing forwards
Agitation	Ewe rapidly walks in circles or back and forward, with the head held above the level of the shoulders, frequently accompanied by high-pitched bleating
Standing/walking	Ewe is maintained on all 4 legs either moving or stationary
Lying	Ewe lies either inactive or ruminating
Eating	Ewe has head within 5 cm of food trough, bucket or hay rack, biting, chewing or pulling hay

### Statistical Analysis

#### Lamb behaviour

Four composite pain variables were calculated as follows: Active Pain Behaviour as the summation of the behaviours footstamp/kick/ease quarters, head-turning, and rolling; Active Pain Behaviour including tail wagging; the composite REQ (as defined in [14]) of Active Pain Behaviours plus restlessness, and REW as a composite of Active Pain Behaviours, tail wagging and restlessness. Lamb behavioural data were not normally distributed therefore all variables were transformed by square root transformations to achieve normality prior to analysis. Data were analysed using linear mixed models (Restricted Maximum Likelihood, REML) in GenStat 11.0 fitting ewe parity, litter size at birth, sire breed and treatment as the fixed effects and ewe identity as a random effect as some lambs in the data set were siblings. Within twin litters, whether the lamb had a male or female twin, or was the first or second in a pair of twin lambs to be treated, was found to have no effect and therefore was dropped from the model. Lamb birth weight was fitted as a covariate in some analyses (Active Pain Behaviour, Active Pain Behaviour + tail wag, REW) where a litter size effect was detected to investigate whether this was related to weight or to some other aspect of birth litter size. Post-hoc pairwise comparisons were made, using Tukey Tests, to determine treatment differences for variables where the model indicated significant effects. Comparisons between litter mates for teat seeking behaviour was made by paired t-tests on transformed data conducted separately for each treatment group to allow the potential effect of treatment on differential lamb behaviour to be discriminated.

#### Maternal behaviour

Data that were not normally distributed were transformed by square root transformations (duration of standing/walking, frequency sniffing/licking lamb, frequency orienting towards lamb) prior to analysis. The effect of lamb treatment on maternal behaviour was analysed using REML, fitting ewe parity, litter size and lamb treatment as fixed effects, and ewe identity as random effects as ewes rearing two lambs had more than one record in the dataset. Treatment differences for significant effects were determined by Tukey tests for pairwise comparisons. For comparisons within ewe to investigate how maternal attention was directed towards the treated and untreated lamb in a pair, Wilcoxon match pairs signed rank tests were used in Minitab 16. Each treatment group were tested separately to determine if ewes responded differently to differing lamb castration methods. To investigate the relationship between lamb pain behaviours and ewe maternal responses Spearman’s rank correlations were calculated.

#### Ethics Statement

This study was conducted under a Project Licence (60/4081) granted by the UK Home Office to C.M.D. under the Animal Scientific Procedures Act (1986). All personnel carrying out procedures on the animals were covered by Personal Licences granted by the Home Office. The entire project was reviewed and approved by the SRUC Animals and Ethics Committee.

## Results

### Lamb Posture

Castration, and method of castration, was associated with an increase in the amount of time that lambs spent in abnormal lateral (Table 3, P<0.001) and ventral lying (P<0.001), and abnormal standing (P<0.001), and a decrease in the amount of time spent in normal lying (P = 0.007), although did not affect time spent in normal standing (Table 3, P = 0.162). RR lambs spent significantly more time in abnormal lateral lying than any other lamb group, and RR and SSC lambs were more likely to be in abnormal ventral lying postures than C or COMBINED lambs. All castrated lambs spent significantly more time in abnormal standing postures than C lambs.

**Table 3:** The effect of different methods of castration on the proportion of time spent by lambs in abnormal and normal standing and lying postures in the 30 minutes following treatment.

Posture	C	RR	SSC	Combined	P (d.f. = 3)
**Abnormal lateral lying**	0.001^a^(0.001–0.01)	0.27^b^(0.167–0.30)	0.009^a^(0.007–0.03)	0.017^a^(0.003–0.04)	W = 50.83P<0.001
**Abnormal ventral lying**	0.000^a^(0.00–0.00)	0.004^b^(0.002–0.007)	0.005^b^(0.002–0.008)	0.000^a^(0.000–0.001)	W = 28.64P<0.001
**Abnormal standing**	0.008^a^(0.001–0.02)	0.083^b^(0.056–0.12)	0.069^b^(0.044–0.10)	0.064^b^(0.040–0.093)	W = 20.20P<0.001
**Normal lying**	0.715^a^(0.619–0.818)	0.409^b^(0.337–0.488)	0.558^ab^(0.473–0.649)	0.627^a^(0.537–0.723)	W = 13.62P = 0.007
**Normal standing**	0.153(0.110–0.203)	0.111(0.075–0.153)	0.194(0.146–0.250)	0.102(0.068–0.143)	W = 5.37P = 0.162

Values are back transformed means with upper and lower interquartile range. Significance was determined with linear mixed models (REML, Wald statistics given) and post-hoc Tukeys tests, within rows means with differing superscripts are significantly different.

### Lamb Behaviour

**Fig 1:**
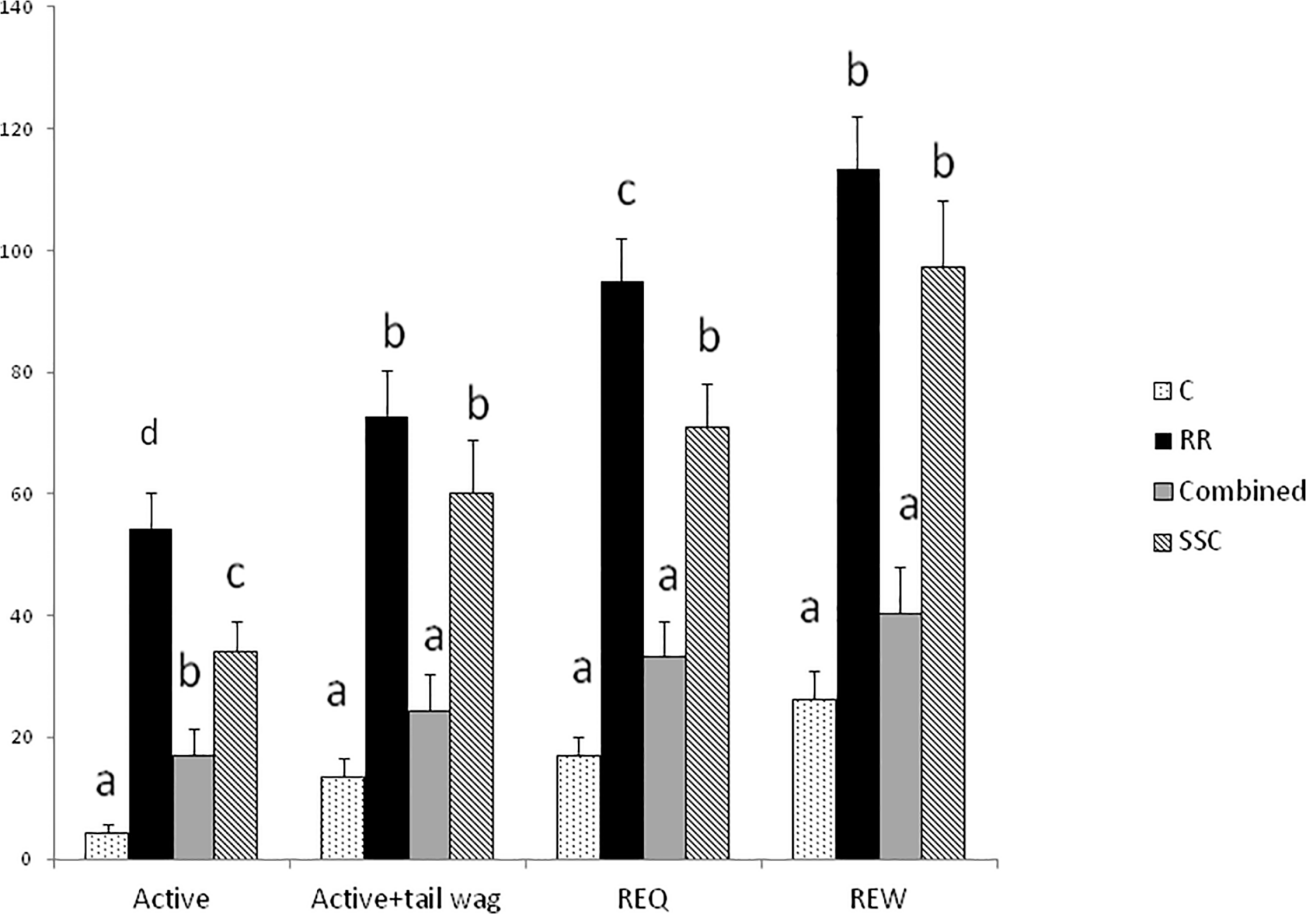
Composite pain behaviours expressed by lambs after different methods of castration. Mean frequency (back transformed with upper 95% confidence interval) of four composite pain behaviours expressed during 30 minutes following handling (C), castration with rubber rings (RR), castration with tight rubber rings followed by Burdizzo (Combined) or short scrotum castration (SSC). Within composite behaviours, treatment groups differ significantly where indicated by differing letters.

Castration treatment had a significant impact on all putative pain-related behaviours, and on the teat seeking response of the treated lamb, but not its untreated sibling (Table 4). In general, RR and SSC lambs showed greater pain associated behaviours than C and COMBINED lambs. Rolling behaviour was seen infrequently but all lambs that rolled after treatment were in the RR group (n = 6/20, 30%). The composite Active Pain Behaviour was significantly different between each castration treatment (Wald = 69.3, d.f. = 3, P<0.001; Fig 1). The other composite measures (Active Pain + tail wag, REQ, REW) derived from the summation of a number of pain-related behaviour as described in Table 2, were significantly greater in RR and SSC lambs than C and COMBINED groups (Wald = 63.32, P<0.001; Wald = 107.23, P<0.001; Wald = 89.07, P<0.001 respectively, d.f. = 3 throughout; Fig 1), with RR also being greater than SSC for Active Pain and REQ behaviours (Fig 1). However, SSC lambs and C lambs showed greater teat-seeking behaviour than COMBINED and RR lambs (Table 4). For SSC and C lambs only, within litter, castrated lambs had a higher teat-seeking frequency than sibling lambs (L2, SSC: Paired T = 3.79, n = 16, P<0.001; C: T = 3.02, n = 16, P = 0.007), but this was not significant in other treatment groups.

**Table 4:** The effect of different methods of castration on the expression of lamb pain-related behaviours in the first 30 minutes following treatment.

Behaviour	C	RR	SSC	Combined	P (d.f. = 3)	
**Footstamp/ kick/ease quarters**	1.48^a^(0.37–3.31)	47.36^d^(39.41–56.05)	26.98^c^(21.06–33.62)	8.47^b^(5.32–12.35)	W = 105.85P<0.001	
**Head turn**	0.66^a^ (0.17–1.47)	8.00^c^(5.91–10.41)	8.51^c^(6.35–10.99)	2.51^b^(1.41–3.93)	W = 40.54P<0.001	
**Tail wag**	8.54^a^(5.45–12.32)	17.12^ab^(12.60–22.33)	25.40^b^(19.82–31.67)	9.64^a^(6.34–13.64)	W = 16.84P = 0.002	
**Restlessness**	9.65^a^(7.34–12.29)	36.24^b^(31.60–41.20)	28.13^b^(24.06–32.52)	12.32^a^(9.68–15.28)	W = 74.62P<0.001	
**Teat seeking (L1)**	1.41^a^(0.86–2.10)	0.14^b^(0.01–0.40)	1.49^a^(0.92–2.19)	0.07^b^(0.00–0.28)		W = 23.45P<0.001
**Teat seeking (L2)**	0.27(0.05–0.67)	0.37(0.10–0.83)	0.25(0.04–0.64)	0.22(0.03–0.59)		W = 0.25NS
**% time spent teat seeking (L1)**	1.81^b^(0.87–3.07)	0.09^c^(0.01–0.50)	3.01^a^(1.76–4.59)	0.05^c^(0.03–0.40)	W = 20.77P<0.001	

Values are back-transformed means (with interquartile ranges) for the frequency of behavioural expression during the 30 minute observation period, or proportion of 30 minute observation spent teat seeking. Significance was determined with linear mixed models (REML, Wald statistics given) and post-hoc Tukeys tests, within rows means with differing superscripts are significantly different.

Sire breed had no significant impact on individual lamb pain behaviours, although there was a tendency for Texel-sired lambs to show greater restlessness than Suffolk-sired lambs (mean frequency in 30 minutes [Q1-Q3]: Texel-sired = 27.3 [19.6–36.3]; Suffolk-sired = 14.0 [8.66–20.7], Wald = 3.41, d.f. = 1, P = 0.069). Single-born lambs tended to have greater footstamp/kick/easing quarters (Wald = 5.99, d.f. = 2, P = 0.058) and had significantly higher tail wagging behaviours (Wald = 17.53, d.f. = 2, P<0.001), and greater composite pain scores, except Active Pain Behaviour (Wald = 5.0, d.f. = 2, P = 0.092) and REQ, than twin or triplet-born lambs (Active Pain Behaviour + tail wag: Wald = 9.44, d.f. = 2, P = 0.013; REW: Wald = 7.69, d.f. = 2, P = 0.028). Fitting lamb birth weight to these analyses suggested that heavier lambs showed a greater frequency of pain behaviours than lighter lambs (effect = 0.69±0.31, P<0.05), but this did not account for the significantly greater pain responses of single lambs compared to twin or triplet lambs. Ewe parity had no significant effects on any measures.

### Maternal Behaviour

**Fig 2:**
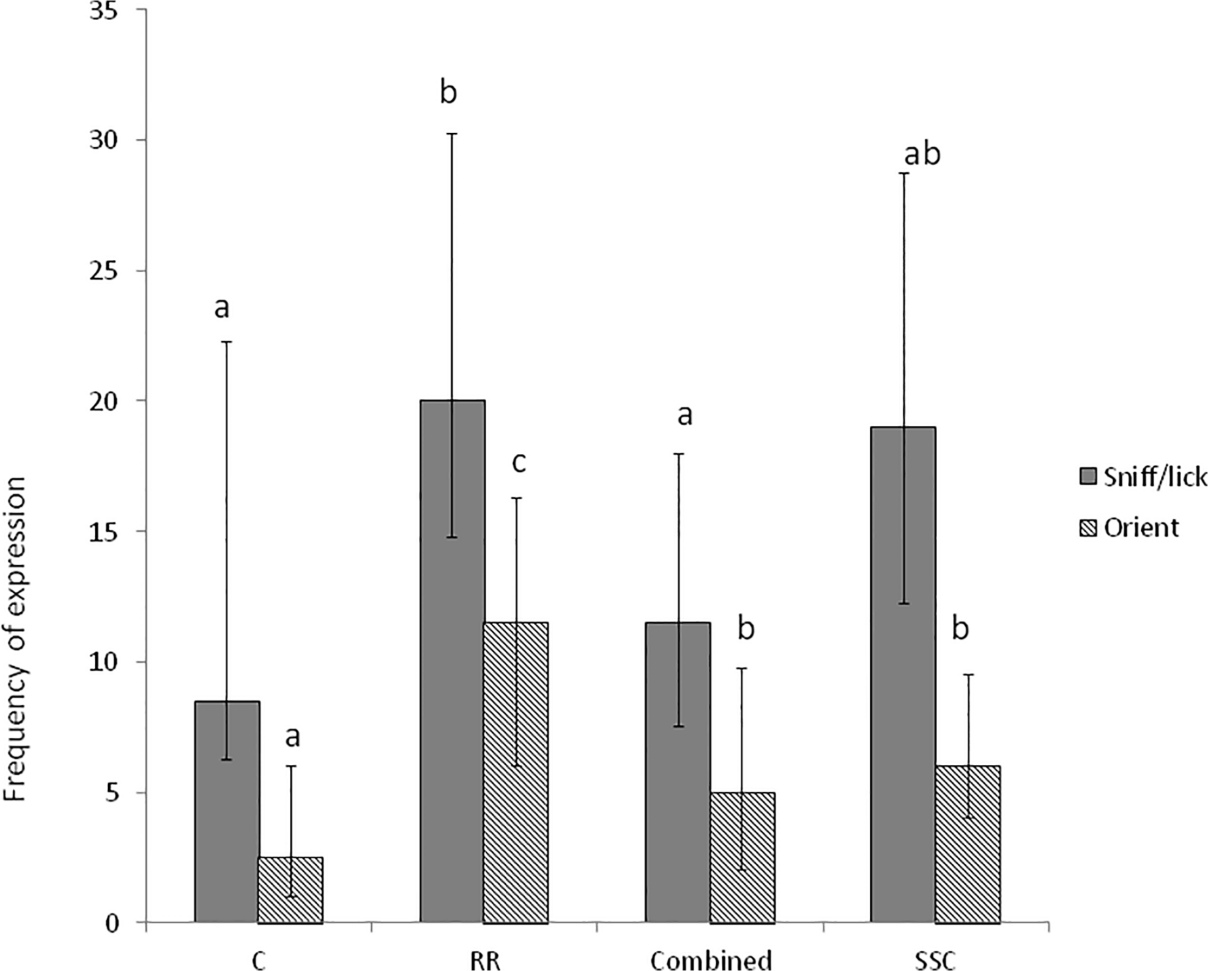
Maternal licking and sniffing behavioural responses. Median frequency (with upper and lower quartile ranges) of maternal sniffing/licking behaviour (solid bars) and orientation (hatched bars) towards her treated lamb (n = 80) by ewes for the 30 minutes following handling (C), castration with rubber rings (RR), castration with tight rubber rings followed by Burdizzo (Combined) or short scrotum castration (SSC) of her lamb. Differences between treatments (within a behaviour) are indicated by different letters.

**Fig 3:**
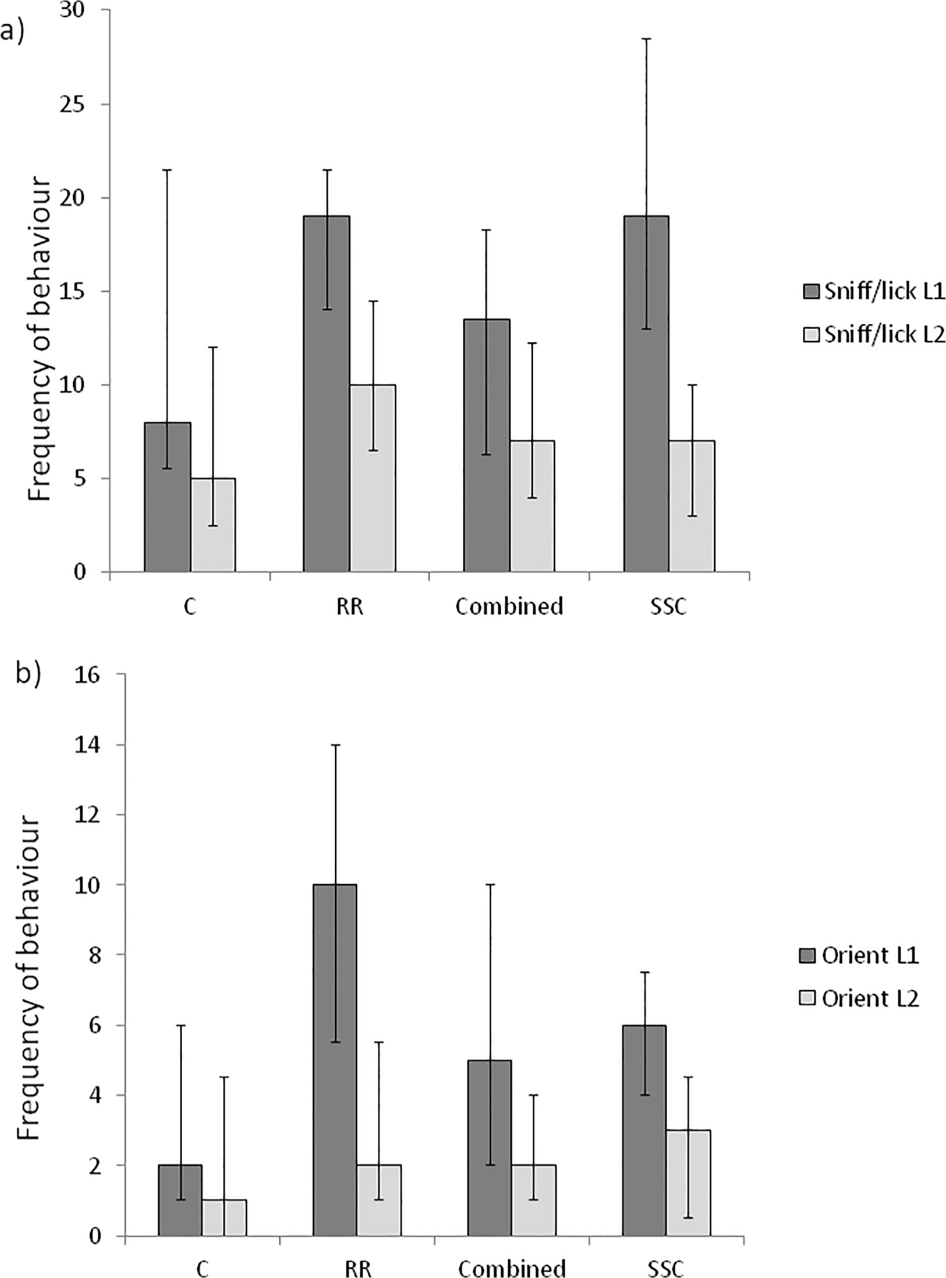
Maternal behaviour expressed towards treated and untreated lambs. Median frequency (with upper and lower quartile ranges) for (a) maternal sniffing/licking and (b) orientation towards the treated lamb (lamb 1, dark grey bars) or the untreated littermate (lamb 2, light grey bars) for 54 ewes with 2 lambs present, for 30 minutes following handling (C), castration with rubber rings (RR), castration with tight rubber rings followed by Burdizzo (Combined) or short scrotum castration (SSC) of lamb 1.

Lamb castration treatment had no significant effects on the amount of time that the ewe spent standing, walking, lying or eating, or on the frequency of ‘head up’ vigilance postures (data not shown). Lamb castration treatment significantly affected ewe sniffing and licking behaviour directed towards the treated lamb (Fig 2, Wald = 9.68, d.f. = 3, P = 0.031), and her orientation towards this lamb (Fig 2, Wald = 26.42, d.f. = 3, P<0.001). Ewes sniffed and licked lambs that had received the treatments RR and SSC significantly more frequently than C or COMBINED lambs. Ewes also oriented towards RR lambs more frequently, and C lambs less frequently, than any other treatment group; ewes oriented towards COMBINED and SSC groups less frequently than RR but more so than towards C lambs. For those ewes rearing two lambs (n = 54) the difference in response was specific to the treated lamb as there were no significant effects of lamb castration treatment on ewe responsiveness towards the untreated litter mate (Fig 3).

Overall, combining all treatments, ewes rearing two lambs directed significantly more sniffing and licking behaviour (W = 2057.0, P<0.001) and oriented more (W = 1652.0, P<0.001) towards the treated than the untreated lamb during the observation period. Within treatment groups C ewes were more likely to sniff/lick the handled lamb than the untreated lamb (Fig 3, W = 102.5, P = 0.002) but did not look any more frequently towards this lamb than its litter mate (W = 67.0, P = 0.142). In all the castrated groups, ewes clearly distinguished between the castrated and the untreated lamb in their sniffing/licking behaviour (Fig 3, RR: W = 146.5, P = 0.001; COMBINED: W = 120.0, P = 0.008; SSC: W = 152.0, P<0.001) and orientation (Fig 3, RR: W = 136.0, P<0.001; COMBINED: W = 97.5, P = 0.005; SSC: W = 108.0, P = 0.007).

### Relationships between ewe and lamb behaviour

There were significant positive correlations between ewe sniffing and licking behaviours and lamb composite pain behavioural responses, and abnormal postures but not the frequency of lamb teat seeking behaviour (Table 5). Similarly, ewes oriented significantly more often towards lambs showing the most active composite pain responses and the greatest amount of time in abnormal standing and lying postures, but not to those showing the highest frequency of teat seeking behaviour.

**Table 5:** Spearman’s rank correlation between frequency of ewe maternal behaviour and lamb behaviours indicative of pain.

Lamb behaviour	Ewe sniffing and licking	Ewe orientation
Abnormal ventral lying	r_s_ = 0.194, P = 0.084	r_s_ = 0.256, P = 0.022
Abnormal lateral lying	r_s_ = 0.312, P = 0.005	r_s_ = 0.541, P<0.001
Abnormal standing	r_s_ = 0.308, P = 0.005	r_s_ = 0.464, P<0.001
Active Pain Behaviour	r_s_ = 0.403, P<0.001	r_s_ = 0.579, P<0.001
Active Pain Behaviour + tail wag	r_s_ = 0.487, P<0.001	r_s_ = 0.540, P<0.001
Restlessness	r_s_ = 0.482, P<0.001	r_s_ = 0.524, P<0.001
REQ	r_s_ = 0.490, P<0.001	r_s_ = 0.592, P<0.001
REW	r_s_ = 0.527, P<0.001	r_s_ = 0.563, P<0.001
Teat-seeking (L1)	r_s_ = 0.071, P = 0.534	r_s_ = -0.161, P = 0.155

Lamb behaviours are proportion of time spent in different postures and frequency of restlessness, composite pain behaviours, as described in Table 2, and teat-seeking responses. N = 80 throughout.

## Discussion

Our study confirmed previous observations [14,16,22] that different castration methods cause different amounts of pain-related behavioural responses from lambs, and that the RR treatment caused the greatest active pain response and abnormal lying in comparison to other methods. We also extend the observations of [17] by demonstrating that ewes not only respond to the pain behaviour of their lambs, but show graded responses in relation to the degree of pain-related behaviour shown by their lambs, and distinguish between littermates that are in pain and those that are not. Twin-rearing ewes whose lambs were handled only, and not subjected to painful stimuli, were also more attentive to their handled lamb than the unhandled littermate, suggesting that these ewes were responding, to some degree, to the manipulation of their offspring. However, there was a quantitative difference in the amount of attention that ewes showed to lambs that had experienced pain, compared to those that had not.

The behaviours and postures used in this study were based on previous studies that defined and validated the behavioural pain responses of lambs to the castration and tail docking [14,16,22,23]. RR and SSC castration methods resulted in the greatest expression of active pain-related behaviour, although RR lambs were the only group to express rolling behaviour associated with severe pain [19] and RR lambs spent more time in abnormal lateral lying than other groups. This suggested that the severity of pain associated with RR castration was greater than SSC where the scrotum and not the testes were within the tissues distal to the ring. COMBINED lambs showed a similar frequency of active pain behaviours to uncastrated C lambs, although the COMBINED group expressed a greater amount of abnormal standing than Control lambs, comparable to the other castrated groups. In studies with much older lambs than used in the present study, Burdizzo treatment has been shown to be associated with an immediate severe response at the point of castration compared to a less severe response in the RR treatment [18]. However, pain associated with RR tends to increase over the first 15 minutes following application of the ring, as the ring gradually contracts and constricts blood flow, so the time course of pain responses differs between treatments. On the basis of their overall active behavioural responses to pain over the 30 minute recording period, it seems that the RR method was the most painful, followed by SSC, and then COMBINED. This ranking also reflects that of [14] based on behaviour and plasma cortisol responses of lambs. We cannot, however, rule out that the COMBINED lambs also experienced pain but this was expressed in a more passive way than the RR or SSC treatment (as suggested by [16,18].

Ewes expressed most sniffing, licking and orienting attention towards RR lambs, followed by SSC, then COMBINED and then Control offspring. Thus, the behaviour of the ewe towards her treated lamb reflected the intensity of the pain-related behavioural responses of the lamb. There was also a moderate positive correlation between ewe attention and lamb abnormal postures and active pain responses, which suggests the greater behavioural activity and abnormality of response attracts the ewes’ attention. Ewes did not, however, show any other signs of behavioural disturbance (e.g. greater activity or vigilance) when their lambs were in pain, which might suggest that they did not experience greater generalised anxiety by the increased pain responses of their lambs compared to handling of their lambs alone.

Control and SSC lambs sought the teat more frequently than their sibling lambs in the 30 minutes following handling or castration but this was not seen in the RR and COMBINED lambs. This may suggest that C and SSC lambs were seeking reassurance from their mothers, following handling or treatment, but does not account for the behaviour of the RR and COMBINED lambs, which did not increase their teat seeking behaviour. It is possible that the RR and COMBINED lambs experienced greater pain and were so unable to express increased teat seeking, although their rate of teat seeking was equivalent to that of their siblings so this seems unlikely. However, the data do show that lambs in pain did not appear to solicit more maternal care from the ewes. Studies in other species have suggested that suckling can provide an analgesic effect. In rats and humans, suckling (both nutritive and non-nutritive) has been shown to reduce the expression of pain-related behaviours [24,25], as does oral administration of sucrose solution [24,26]. In farm animals, however, neither sucrose infusion nor suckling has been shown to be effective in reducing the pain of castration and/or tail docking in pigs or sheep [27–29]. This may be due to a greater intensity of the pain stimulus experienced in these species, or a different type of pain induced by these management procedures, which seems to inhibit teat-seeking and eliminate any analgesic influence of ingestion of sweet solutions. The increase in teat seeking behaviour of the Control lambs, compared to their untreated littermates, does suggest that this may exert a calming influence on lambs that are not in pain, following the mild stress of being handled. However, the increase in teat seeking behaviour in the SSC group is intriguing, and may be related to a different quality of pain in this group compared to the other castration groups, although it was anticipated that pain in this group would be similar to but less intense compared to the RR group from previous studies [14].

In studies of humans and rats increased maternal care of the offspring experiencing pain reduced pain expression [5,30]. In human babies undergoing painful hospital procedures maternal skin-to-skin contact significantly reduced heart rate, crying and grimacing [31]. In sheep, Hild and colleagues [9] demonstrated that lambs that stayed closest to their mother, and were more synchronised with her behaviour, had a higher nociceptive threshold, i.e. lower pain sensitivity, as measured by a thermal stimulus, in comparison to lambs that were further from their mothers. This suggests that in sheep, as in rodents and humans, increased maternal care can reduce the expression of pain. Whether the increased maternal care elicited by lamb pain responses in the present study acted to reduce the pain sensitivity of the lambs is unclear, as the greatest maternal attention was directed towards the lambs expressing the most pain-related behaviours. Separation of the ewe and lamb at castration, or prevention of expression of maternal behaviour, may be required to understand if maternal care plays a role in reducing pain expression in castrated lambs, and whether this can influence the expression of pain sensitivity in later life.

Our data also suggest that lamb weight, litter size and sire breed have some influence on the responses of the lamb to castration treatment. The effect of lamb weight can be possibly be explained by the volume of tissue retained within the rubber ring as all castration treatments involved the use of a rubber ring. The time taken by the ring to shrink back to its original shape, which then induces tissue necropsy and nerve death, will be influenced by the amount and resistance of the soft tissues within the ring (as previously discussed [32]), thus larger lambs, with larger volumes of scrotal tissue will experience slower nerve death. However single lambs displayed greater pain-related behaviour than multiple-born lambs, which was not completely accounted for by their heavier birth weight. It is possible that the presence of an untreated sibling, in multiple litters, may act as social support for the treated lamb, in addition to its mother, as has been described for the stress of maternal separation [33]. In a study of unrelated lambs, Colditz and colleagues [34] concluded that there was no evidence of social support for castrated lambs, or of emotional contagion, by the presence of uncastrated lambs. However, there is some evidence that social buffering by a familiar twin lamb does occur with tail docking and that this is more effective than the presence of an unfamiliar or unrelated [35], and this influence may contribute to the litter effects seen in the present study. The influence of breed on behaviour of castrated lambs has been reported previously [20], as was found here, although this may reflect a different pattern of response rather than altered nociceptive thresholds.

## Conclusions

Maternal ewes are sensitive to the pain expression of their offspring, and direct most maternal care towards their offspring exhibiting the most extreme pain-related behaviours. Whether this is a response of the ewe to the clearly abnormal behaviour of the lamb which attracts her attention, or related to some other empathetic response of the ewe is not clear. In other species this additional maternal care appears to act as a buffer to reduce pain responses, and may reduce the sensitisation of the neonate to later pain exposure. However, whether similar responses occur in neonatal lambs is not known.

## Supporting Information

S1 Table The data on which this article is based are provided in an excel file. (XLSX)

## References

[1] Dwyer CM (2014) Maternal behaviour and lamb survival: From neuroendocrinology to practical application. Animal 2014;8: 102–112. 10.1017/S1751731113001614 24103485

[2] Walker C-D. Maternal touch and feed as critical regulators of behavioral and stress responses in the offspring. Dev Psychobiol. 2010;52: 638–650. 10.1002/dev.20492 20862707

[3] Johnston C, Campbell-Yeo M, Fernandes A, Inglis D, Streiner D, Zee R. Skin-to-skin care for procedural pain in neonates. Cochrane Database of Systematic Reviews 2014;CD008435, 10.1002/14651858.CD008435.pub2 24459000

[4] Blass EM, Shide DJ, Zawmon C, Sorrentino J. Mother as shield–differential effects of contact and nursing on pain responsivity in infant rats–evidence for non opioid mediation. Behav Neurosci. 1995;109: 342–353. 7619324 10.1037//0735-7044.109.2.342

[5] Walker C-D, Kudreikis K, Sherrard A, Johnston CC. Repeated neonatal pain influences maternal behavior, but not stress responsiveness in rat offspring. Dev Brain Res 2003;140: 253–261. 12586430 10.1016/s0165-3806(02)00611-9

[6] Chung EKY, Zhang XJ, Li Z, Zhang HQ, Xu HX, Bian ZX. Neonatal maternal separation enhances central sensitivity to noxious colorectal distention in rat. Brain Res. 2007;1153: 68–77. 17434464 10.1016/j.brainres.2007.03.047

[7] Weaver SA, Diorio J, Meaney MJ. Maternal separation leads to persistent reductions in pain sensitivity in female rats. J. Pain 2007;8, 962–969. 17686657 10.1016/j.jpain.2007.07.001

[8] de Medeiros CB, Fleming AS, Johnston CC, Walker C-D. Artificial rearing of rat pups reveals the beneficial effects of mother care on neonatal inflammation and adult sensitivity to pain. Pediatr Res. 2009;66: 272–277. 10.1203/PDR.0b013e3181b1be06 19531973

[9] Hild S, Andersen IL, Zanella AJ. The relationship between thermal nociceptive threshold in lambs and ewe-lamb interactions. Small Rum Res. 2010;90: 142–145.

[10] Clark C, Murrell J, Fernyhough M, O'Rourke T, Mendl M. Long-term and trans-generational effects of neonatal experience on sheep behaviour. Biol Lett. 2014;10 Article Number: 20140273, 10.1098/rsbl.2014.0273 PMC4126620 25115031

[11] Jewell PA. Survival and behaviour of castrated Soay sheep (Ovis aries) in a feral island population on Hirta, St Kilda, Scotland. J Zool. 1997;243: 623–636.

[12] Kemp JD, Cruse JD, DeWeese W, Moody WG. (1970) Effect of slaughter weight and castration on carcass characteristics of lambs. J Anim Sci. 1970;30: 348 5461738 10.2527/jas1970.303348x

[13] Farm Animal Welfare Council (2008) FAWC report on the implications of castration and tail docking for the welfare of lambs. Accessed June 2015: https://www.gov.uk/government/uploads/system/uploads/attachment_data/file/325125/FAWC_report_on_the_implications_of_castration_and_tail_docking_for_the_welfare_of_lambs.pdf

[14] Molony V, Kent JE, McKendrick IJ. Validation of a method for the assessment of an acute pain in lambs. Appl Anim Behav Sci. 2002;76: 215–238.

[15] Molony V, Kent JE. Sheep welfare: castration and tail docking. In: Aitken ID, editor. Diseases of sheep, Blackwell. 2007. pp. 27–32.

[16] Mellema SC, Doherr MG, Wechsler B, Thuer S, Steiner A. Influence of local anaesthesia on pain and distress induced by two bloodless castration methods in young lambs. Vet J. 2006;172: 274–283. 16051508 10.1016/j.tvjl.2005.06.002

[17] Hild S, Clark CCA, Dwyer CM, Murrell JC, Mendl M, Zanella AJ. Ewes are more attentive to their offspring experiencing pain but not stress. Appl Anim Behav Sci. 2011;132: 114–120.

[18] Melches S, Mellema SC, Doherr MG, Wechsler B, Steiner A. Castration of lambs: A welfare comparison of different castration techniques in lambs over 10 weeks of age. Vet J. 2007;173: 554–563. 16527503 10.1016/j.tvjl.2006.01.006

[19] Molony V, Kent JE, Robertson IS. Behavioural responses of lambs of 3 ages in the first 3 hours after 3 methods of castration and tail docking. Res Vet Sci. 1993;55: 236–245. 8235093 10.1016/0034-5288(93)90087-v

[20] Archer N, Johnston AM, Khalid M. Differences in the acute pain responses of two breeds of lamb following castration and tail docking with the rubber ring method. Anim Welfare 2004;13: 135–141.

[21] Pickup HE, Dwyer CM. (2011) Breed differences in the expression of maternal care at parturition persist throughout the lactation period in sheep. Appl Anim Behav Sci. 2011;132: 33–41.

[22] Molony V, Kent JE. Assessments of acute pain in farm animals using behavioural and physiological measurements. J Anim Sci. 1997;75: 266–272. 9027575 10.2527/1997.751266x

[23] Kent JE, Molony V, Graham MJ. The effect of different bloodless castrators and different tail docking methods on the responses of lambs to the combined Burdizzo rubber ring method of castration. Vet J. 2001;162: 250–254. 11681876 10.1053/tvjl.2001.0598

[24] Blass EM, Watt LD. Suckling- and sucrose-induced analgesia in human newborns. Pain 1999;83: 611–623. 10568870 10.1016/S0304-3959(99)00166-9

[25] Anseloni V, Ren K, Dubner R, Ennis M. Ontogeny of analgesia elicited by non-nutritive suckling in acute and persistent neonatal rat pain models. Pain 2004;109: 507–513. 15157713 10.1016/j.pain.2004.02.031

[26] Ren K, Blass EM, Zhou QQ, Dubner R. Suckling and sucrose ingestion suppress persistent hyperalgesia and spinal Fos expression after forepaw inflammation in infant rats. Proc Nat Acad Sci USA. 1997;94: 1471–1475. 9037077 10.1073/pnas.94.4.1471 PMC19815

[27] Price J, Nolan AM. Analgesia of newborn lambs before castration and tail docking with rubber rings. Vet Record 2001;149: 321–324. 10.1136/vr.149.11.321 11583126

[28] Rand JS, Noonan GJ, Priest J, Ainscow J, Blackshaw JK. Oral administration of a 12% sucrose solution did not decrease behavioural indicators of distress in piglets undergoing tail docking, teeth clipping and ear notching. Anim Welfare 2002;11: 395–404.

[29] Landa L. The effect of milk suckling from the dam or glucose administration on the behavioural responses to tail docking in lambs. Acta Vet Brno 2003;72: 175–182.

[30] Walker C-D, Xu ZF, Rochford J, Johnston CC. Naturally occurring variations in maternal care modulate the effects of repeated neonatal pain on behavioral sensitivity to thermal pain in the adult offspring. Pain 2008;140: 167–176. 10.1016/j.pain.2008.08.004 18801618

[31] Gray L, Watt L, Blass EM. (2000) Skin-to-skin contact is analgesic in healthy newborns. Pediatrics 2000;105: e14 10617751 10.1542/peds.105.1.e14

[32] Molony V, Kent J, Vinuela-Fernandez I, Anderson C, Dwyer CM. Pain in lambs castrated at two days with novel smaller and tighter rubber rings compared with conventional rubber rings and after local anaesthetic treatment. Vet J. 2012;193: 81–86. 10.1016/j.tvjl.2011.09.030 22178156

[33] Porter RH, Nowak R, Orgeur P. (1995) Influence of a conspecific agemate on distress bleating by lambs. Appl Anim Behav Sci. 1995;45: 239–244.

[34] Colditz IG, Paull DR, Lee C. Social transmission of physiological and behavioural responses to castration in suckling Merino lambs. Appl. Anim. Behav. Sci. 2012;136: 136–145.

[35] Guesgen MJ, Beausoleil NJ, Minot E, Stewart M, Stafford KJ. Social context and other factors influence behavioural pain expression by lambs. Appl Anim Behav Sci 2014;159: 41–49.

